# MMP24 as a Target of YAP Is a Potential Prognostic Factor in Cancer Patients

**DOI:** 10.3390/bioengineering7010018

**Published:** 2020-02-20

**Authors:** Wataru Sugimoto, Katsuhiko Itoh, Hiroaki Hirata, Yoshinori Abe, Takeru Torii, Yasumasa Mitsui, Yemima Budirahardja, Nobuyuki Tanaka, Keiko Kawauchi

**Affiliations:** 1 Frontiers of Innovative Research in Science and Technology, Konan University, Kobe 650-0047, Japan; los.wrrrwve.56s2@gmail.com (W.S.); itokatsuuu@gmail.com (K.I.); grignard.tor2@gmail.com (T.T.); dadagagaga15@gmail.com (Y.M.); ybudirahardja@gmail.com (Y.B.); 2 Mechanobiology Laboratory, Nagoya University Graduate School of Medicine, Nagoya 466-8550, Japan; hhirata@med.nagoya-u.ac.jp; 3 Department of Molecular Oncology, Institute for Advanced Medical Sciences, Nippon Medical School, Tokyo 113-0033, Japan; yoshiabe@nms.ac.jp

**Keywords:** MMP, YAP, ECM, mechanical signal transduction, cancer progression, prognosis

## Abstract

The extracellular matrix (ECM) surrounding cancer cells becomes stiffer during tumor progression, which influences cancer cell behaviors such as invasion and proliferation through modulation of gene expression as well as remodeling of the actin cytoskeleton. In this study, we show that *MMP24* encoding matrix metalloproteinase (MMP)-24 is a novel target gene of Yes-associated protein (YAP), a transcription coactivator known as a mechanotransducer. We first examined the effect of substrate stiffness on *MMP24* expression in MCF-7 human breast cancer cells and showed that the expression of *MMP24* was significantly higher in cells grown on stiff substrates than that on soft substrates. The *MMP24* expression was significantly reduced by knockdown of YAP. In contrast, the expression of constitutively active YAP increased *MMP24* promoter activity. In addition, binding of YAP to the *MMP24* promoter was confirmed by the chromatin immunoprecipitation (ChIP) assay. These results show that ECM stiffening promotes YAP activation, thereby inducing *MMP24* expression. Based on the Human Protein Atlas database, breast cancer patients with lower *MMP24* expression exhibit the worse survival rates overall. Thus, *MMP24* may negatively regulate the aggressiveness of cancer cells under the stiff ECM environment during tumor progression.

## 1. Introduction

The mechanical properties of the extracellular matrix (ECM) surrounding cancer cells are altered in the process of tumor progression. Secretion of ECM proteins by cancer-associated fibroblasts and myofibroblasts in the tumor microenvironment causes stiffening of the ECM [1,2]. The increase in ECM stiffness modulates cancer cell behaviors via the alteration of both migration and gene expression, wherein remodeling of the actin cytoskeleton plays an integral role. Binding of the ECM to integrins, transmembrane receptors for ECM proteins, activates Rho GTPases and thereby promotes actin polymerization and myosin activation [3,4,5,6]. Rigid ECM enhances this integrin signaling, leading to an increase in actin filament formation. Interaction of Yes-associated protein (YAP) and angiomotin (AMOT), which sequesters YAP in the cytoplasm, is attenuated by F-actin association to AMOT, resulting in the nuclear translocation of YAP [7,8]. Our previous study using MCF-7 cells has shown that an increase in actomyosin activity upregulates YAP-dependent gene expression [9,10].

Matrix metalloproteinases (MMPs) belong to a family of zinc-dependent endopeptidases and are categorized into two distinct classes: soluble MMPs and membrane-type (MT) MMPs [11]. MT-MMPs cleave not only the ECM but also other substrates including prototype MMPs [12]. Substrates of MMP24, also known as MT5-MMP, include ECM proteins such as gelatin and fibronectin, N-cadherin, amyloid precursor protein and MMP2 [12,13,14]. MMP24 is dominantly expressed in neuronal cells and the function of MMP24 in Alzheimer’s disease has been well studied. However, little is known about the role of MMP24 in cancer—particularly breast cancer.

Here, we showed that substrate stiffening enhances YAP-dependent *MMP24* expression through the activation of actomyosin contraction. Breast cancer patients with lower *MMP24* expression show lower survival rates. Considering that cancer cell behaviors such as invasion and proliferation are often promoted in response to rigid substrates, MMP24 is likely to act as a negative regulator for cancer progression.

## 2. Materials and Methods 

### 2.1. Cell Culture and Materials

MCF-7 human breast cancer cells and 293T human embryonic kidney cells were cultured in Dulbecco’s modified Eagle’s medium (Nissui Pharmaceutical, Tokyo, Japan) supplemented with 10% fetal bovine serum and 1% penicillin/streptomycin at 37 °C under 5% CO_2_. N-acryloyl-6-aminocaproic acid-copolymerized acrylamide gels for polyacrylamide culture substrates were prepared as described previously [15,16]. Latrunculin A and blebbistatin was purchased from Sigma-Aldrich (St. Louis, MO, USA) and Merck Millipore (Burlington, MA, USA), respectively. An 8× GTIIC-luciferase plasmid obtained from Addgene contains 8× TEAD binding sites. pCS2-YAP 5SA, a gift from Dr. Hiroshi Nishina, expresses a constitutive active form of YAP, and contains the insert phosphorylation-defective YAP 5SA mutant. 

### 2.2. Retroviral Vectors and Retroviral Infection

The retrovirus vectors encoding small hairpin RNAs (shRNAs) against human *YAP* and human *ROCK2*, the *YAP* target sequences #1: 5′-GCCACCAAGCTAGATAAAGAA-3′, #2: 5′-GACATCTTCTGGTCAGAGA-3′ and *ROCK2* target sequence: 5′-GGTTTATGCTATGAAGCTT-3′, were cloned into the pSuper retro puro vector (Oligoengine, Seattle, WA, USA) and pSuper retro hygro vector [10], respectively. Retroviral infection was performed as described previously [16]. The cells were selected using puromycin (1.5 μg/mL) or hygromycin (300 μg/mL) for 3 days.

### 2.3. Luciferase Assay

The reporter construct MMP24-luc was generated by sub-cloning the PCR-amplified fragment encompassing the promoter region (−1010 to +3) of the human *MMP24* gene into the pGL3-basic vector. The control plasmid phRL-TK (Renilla luciferase reporter) was obtained from Toyobo (Osaka, Japan). Luciferase activity was determined using the Dual-Luciferase Reporter Assay System (Promega, Madison, WI, USA).

### 2.4. Quantitative Real-Time PCR

Total RNA was purified using NucleoSpin RNA kit (Takara Bio Inc., Shiga, Japan). cDNA was prepared using PrimeScript 1st strand cDNA Synthesis kit (Takara Bio Inc., Shiga, Japan). Quantitative real-time PCR analysis was performed with Thunderbird SYBR qPCR Mix (Toyobo, Osaka, Japan) under the following conditions: 10 min at 95 °C, followed by 40 cycles of 95 °C for 15 s and 55 °C for 1 min using StepOne Plus Real-Time PCR system (Applied Biosystems). The following primers were used: human *YAP* forward 5′-AGGAGAGACTGCGGTTGAAA-3′ and reverse 5′-CCCAGGAGAAGACACTGCAT-3′; human *MMP24* forward 5′-GCAGAAGGTGACCCCACTGA-3′ and reverse 5′-CATTTCCTAGCGTCCATGGC-3′; human *MMP7* forward 5′-GGGCAAAGAGATCCCCCTGCAT-3′ and reverse 5′-CCCAGGCGCAAAGGCATGAG-3′; human *CTGF* forward 5′-ACCGACTGGAAGACACGTTTG-3′ and reverse 5′-CCAGGTCAGCTTCGCAAGG-3′; human *ROCK2* forward 5′-CAACTGTGAGGCTTGTATGAAG-3′ and reverse 5′-TGCAAGGTGCTATAATCTCCTC-3′, and human *ubiquitin* forward 5′-TGACTACAACATCCAGAA-3′ and reverse 5′-ATCTTTGCCTTGACATTC-3′.

### 2.5. Fluorescence Microscopy

Cells were fixed with 4% PFA and then permeabilized with 0.1% Triton X-100. After blocking with 2% BSA in phosphatase buffered saline (PBS), the cells were incubated with the anti-YAP rabbit polyclonal antibody (D8H1X; Cell Signaling Technology, Danvers, MA, USA). Alexa Fluor 488 conjugated goat anti-rabbit IgG (Molecular Probes Carlsbad, CA) was used as a secondary antibody. DAPI (Vector Laboratories, Inc., Burlingame, CA, USA) was used to stain nuclei. Images were acquired using a confocal microscope (LSM700; Zeiss) and then analyzed with ImageJ software (NIH).

### 2.6. Chromatin Immunoprecipitation (ChIP) Assay

The assay was performed as described previously [10]. Cross-linked chromatin was immunoprecipitated with anti-YAP rabbit polyclonal antibody (D8H1X; Cell Signaling Technology, Danvers, MA, USA) or normal rabbit IgG (Cell Signaling Technology, Danvers, MA, USA) antibodies. Precipitated DNA was analyzed by quantitative real-time PCR. The following primers were used to amplify the human *MMP24* promoter region −689 to −460 containing the predicted TEAD-binding sequence: forward 5′-GATCTTCCCAGCTGGATGAGC-3′ and reverse 5′-GTAAAGGCGGGGTTCGAGAG-3′.

### 2.7. ChIP-Seq Database Analysis

Genome-wide occupancy dataset for TEAD4 in MCF-7 cells was obtained from the Encyclopedia of DNA Elements (ENCODE) consortium with track names MCF-7 TEAD4_V11_1 and _2. ChIP analysis was performed using anti-TEAD4 antibody (SantaCruz Biotechnology, sc-101184, Lot A1811) and the ChIP-seq data was mapped to the NCBI GRCh37/hg19 human genome sequence. The ENCODE dataset was visualized using the UCSC genome browser (http://genome.ucsc.edu/index.html).

### 2.8. The Human Protein Atlas Analysis

Correlations between *MMP24* or *MMP7* expression and the survival rate of breast, lung, pancreatic, renal and colorectal cancer patients were analyzed using the Human Protein Atlas database (https://www.proteinatlas.org) [17].

### 2.9. Statistical Analysis

Statistical analysis of data was performed using the unpaired Student’s two-sided *t*-test.

## 3. Results

**Figure 1:**
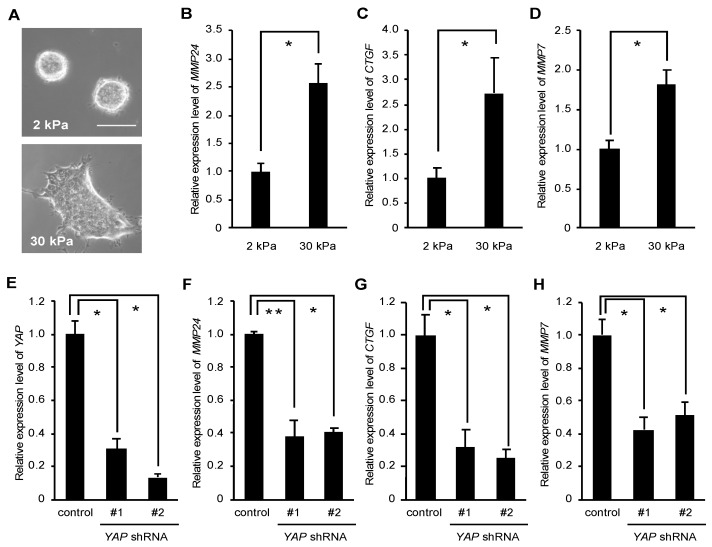
Yes-associated protein (YAP) mediates *MMP24* expression induced by stiffening substrates. (**A**–**D**) MCF-7 cells were cultured on substrates with elasticities of 2 and 30 kPa. Phase contrast images of the cells were obtained with an inverted microscope (Olympus CKX41). Scale bar, 50 μm (**A**). The expression of *MMP24* (**B**), *CTGF* (**C**) and *MMP7* (**D**) were evaluated by quantitative real-time PCR. Each bar represents the mean ± S.D.; n = 3. Asterisks represent *p* < 0.005. (**E**–**H**) Cells were infected with a control, *YAP* shRNA-#1, or -#2-expressing retrovirus. The expression of *YAP* (**E**), *MMP24* (**F**), *CTGF* (**G**), and *MMP7* (**H**) were evaluated by quantitative real-time PCR. Each bar represents the mean ± S.D.; n = 3. Asterisks represent *p* < 0.005. Double asterisk represents *p* < 0.01.

Using a microarray to compare the gene expression profile of T84 human colorectal cancer cells grown on stiff versus soft substrates, Nukuda et al. showed that the expression level of several MMPs including *MMP24* was elevated when cells were cultured on stiff substrates [18]. We wondered whether rigid substrates also promote *MMP24* expression in breast cancer cells. As shown previously, MCF-7 human breast cancer cells cultured on the soft substrate (2 kPa) formed spheroids and those cultured on the rigid substrate (30 kPa) were spread out (Figure 1A), indicating that MCF-7 cells sense and respond to the differential stiffness between 2 kPa and 30 kPa. Using quantitative real time PCR, we observed that the expression level of *MMP24* was significantly higher in cells grown on the rigid substrate compared to those on the soft substrate (Figure 1B). Similarly, the expression levels of two YAP target genes, *CTGF* and *MMP7*, were also elevated in cells grown on the rigid substrate (Figure 1C,D). We have previously shown that rigid substrates promote YAP-dependent transcription in MCF-7 cells [9,10]. We therefore examined whether YAP regulated *MMP24* expression. To this end, we depleted *YAP* expression using two shRNAs against human *YAP* (Figure 1E) and evaluated the expression of *MMP24* and *CTGF*, a well-known YAP target gene, in cells grown on plastic dishes, i.e., rigid substrates (elastic modulus ~10^6^ kPa). We observed that *YAP* knockdown caused ~2.5-fold reduction, ~3.3-fold reduction and ~2-fold reduction in the *MMP24*, *CTGF* and *MMP7* expression levels, respectively (Figure 1F–H).

**Figure 2:**
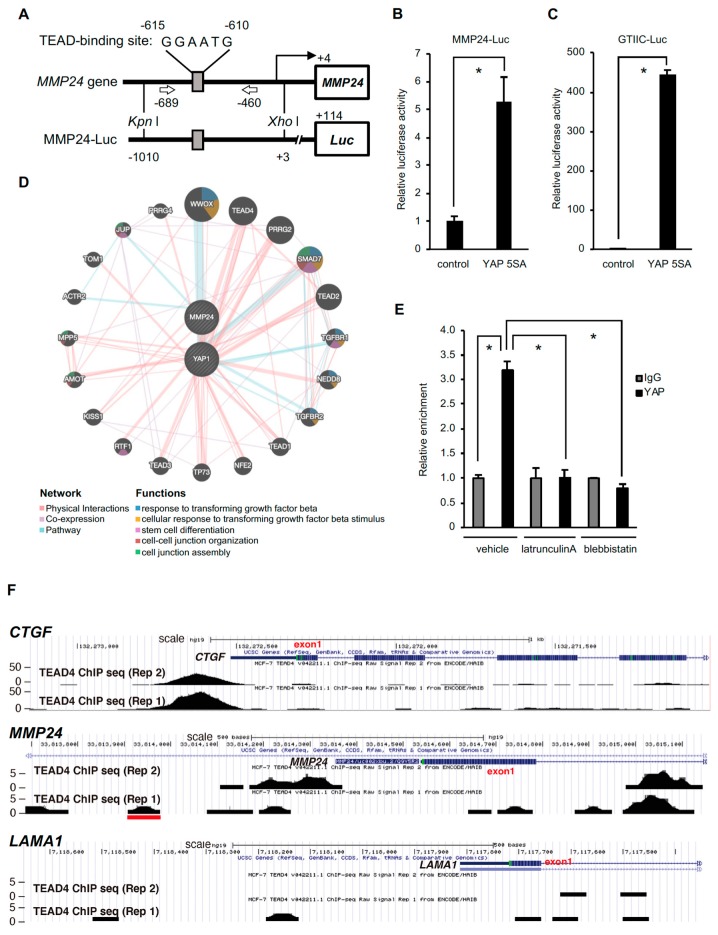
Binding of YAP to the *MMP24* promoter is decreased by inhibition of actomyosin contractility. (**A**) Location of TEAD-binding sequences within the *MMP24* promoter and the luciferase reporter gene construct. TEAD-binding sequences within the *MMP24* promoter are shown (top). The MMP24-Luc reporter plasmid contains the promoter region of the human *MMP24* gene located at position −1010 to +3. (**B**) The pCS2-YAP 5SA expression vector was co-transfected with the reporter plasmid into 293T cells. Luciferase activity was measured 24 hours after transfection. The activity was normalized to that of the control vector. Each bar represents the mean ± S.D.; n = 3. Asterisks represent *p* < 0.05. (**C**) A TEAD-responsive element luciferase reporter plasmid (8× GTIIC-Luc) was used as a control. Each bar represents the mean ± S.D.; n = 3. Asterisks represent *p* < 0.005. (**D**) Predicted network for YAP and MMP24 was created using the GeneMANIA online software at http://www.genemania.org/. A graphic representation of how YAP and MMP24 are related to each other and to other genes in terms of physical interactions, pathways, and co-expression is shown in [19]. (**E**) MCF-7 cells were treated with 200 nM latrunculin A or 50 μM blebbistatin for 40 min. The binding of YAP to the *MMP24* promoter was evaluated by chromatin immunoprecipitation assay followed by quantitative real-time PCR. The position of the primers is indicated by arrows in (**A**). Each bar represents the mean ± S.D.; n = 3. Asterisks represent *p* < 0.005. (**F**) The UCSC Genome Browser (http://genome.ucsc.edu/index.html) results show the locations of TEAD4 chromatin immunoprecipitation (ChIP)-Seq signals on the *CTGF*, *MMP24* or *LAMA1* locus. TEAD4 ChIP-Seq data from MCF-7 cells was obtained from the ENCODE consortium. Two replicated results are shown. The red line indicates the human *MMP24* promoter region (approximately −590 to −650) containing the predicted TEAD-binding sequence.

The *MMP24* gene has the TEA domain (TEAD) recognition sequence GGAATG at −610 to −605 upstream of the transcription start site, and YAP may bind to this sequence through TEAD (Figure 2A). We then constructed a luciferase reporter plasmid containing the region −1010 to +3 of the *MMP24* gene and used it to examine the role of YAP in the *MMP24* promoter activation. Ectopic expression of the constitutive active form of YAP (YAP 5SA) increased the *MMP24* promoter activity (Figure 2B) as well as the TEAD-dependent reporter activity (Figure 2C). In addition, the signal pathway analysis using the public database GeneMANIA suggests a link between *MMP24* and *YAP* (Figure 2D). We then performed ChIP assay to confirm the binding of YAP to the *MMP24* promoter and found that the amount of precipitated DNA of the *MMP24* promoter region (−689 to −460) containing the predicted TEAD-binding sequence, using anti-YAP antibody, was significantly higher than that using anti-IgG antibody as a negative control (Figure 2E). Furthermore, analysis using the UCSC Genome Browser showed that TEAD4 binds to the *CTGF* and *MMP24* promoters but not *LAMINA* promoter, which was used as a negative control (Figure 2F). Peak TEAD4 binding was observed in the region containing the predicted TEAD-binding sequence of the *MMP24* promoter (Figure 2F, middle, red line). All these results indicate that *MMP24* is a target gene of YAP-TEAD. 

Even though the binding of TEAD4 to the *MMP24* promoter was weaker than that to the *CTGF* promoter (Figure 2F), the effect of substrate stiffness or *YAP* knockdown on *MMP24* expression was similar to that on *CTGF* expression (Figure 1B vs. Figure 1C, Figure 1 F vs. Figure 1 G, respectively). Notably, TEAD4 also binds to intron 1 of the *MMP24* gene (Figure 2F, upper vs. middle). Thus, YAP and TEAD may upregulate *MMP24* expression by their binding not only to the promoter but also to intron 1 of *MMP24*.

The nuclear translocation of YAP has been shown to be dependent upon actomyosin contractility [7,8,9,10]. We thus set out to investigate the role of actomyosin in the interaction between YAP and the *MMP24* promoter. Treatment with latrunculin A, an inhibitor of actin polymerization, or blebbistatin, a myosin II inhibitor, diminished the binding of YAP to the *MMP24* promoter (Figure 2E), indicating the importance of actomyosin activity for YAP-dependent *MMP24* expression. 

**Figure 3:**
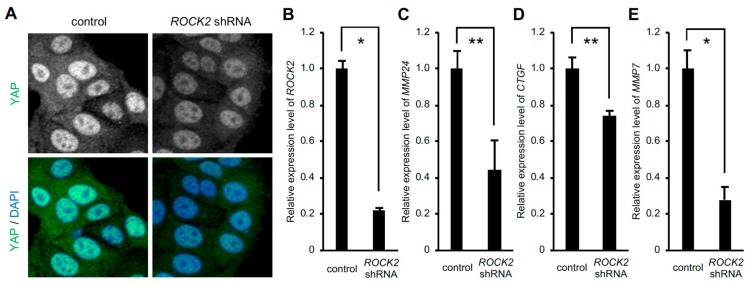
Knockdown of *ROCK2* decreases *MMP24* expression**.** Cells were infected with *ROCK2* shRNA-expressing retrovirus. Confocal images of cells stained for YAP (green) and DAPI (blue) are shown (**A**). The expression of *ROCK2* (**B**), *MMP24* (**C**), *CTGF* (**D**), and *MMP7* (**E**) were evaluated by quantitative real-time PCR. Each bar represents the mean ± S.D.; n = 3. Asterisks represent *p* < 0.005. Double asterisks represent *p* < 0.01.

We have demonstrated that expression of the gene encoding Rho-associated coiled coil-containing protein kinase (ROCK) 2, which induces the activation of myosin II, is increased in response to the rigid substrate in a YAP-TEAD dependent manner [9]. On the other hand, nuclear accumulation of YAP, in turn, depends on *ROCK2* expression, as shown previously [10] and in Figure 3A. Therefore, we wondered whether ROCK2 played a role in the expression of *MMP24* in cells cultured on the rigid substrate. Knockdown of *ROCK2* significantly reduced expression levels of *MMP24* as well as *CTGF* and *MMP7* (Figure 3B–E), indicating that ROCK2 was involved in YAP-dependent *MMP24* expression.

**Figure 4:**
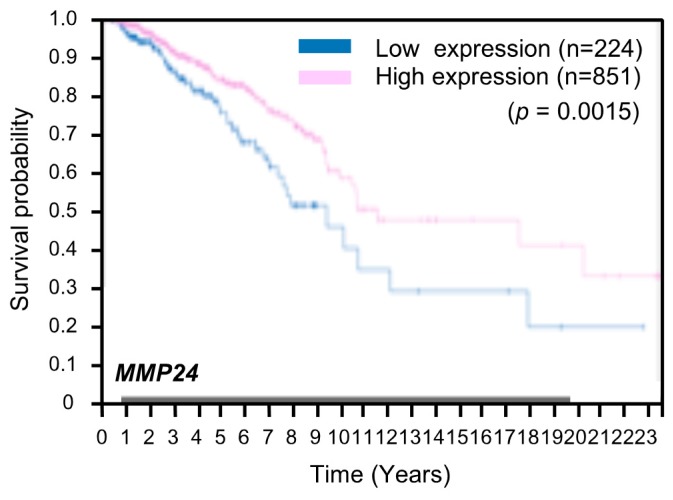
Low expression of *MMP24* predicts poor prognosis in patients with breast cancer. Kaplan–Meier plots for the survival analysis of human breast cancer patients with high (magenta) and low (blue) *MMP24* expression obtained from the Human Protein Atlas (version 19) available at www.proteinatlas.org. The link is: (https://www.proteinatlas.org/ENSG00000125966-MMP24/pathology/breast+cancer#imid_20796312).

**Figure 5:**
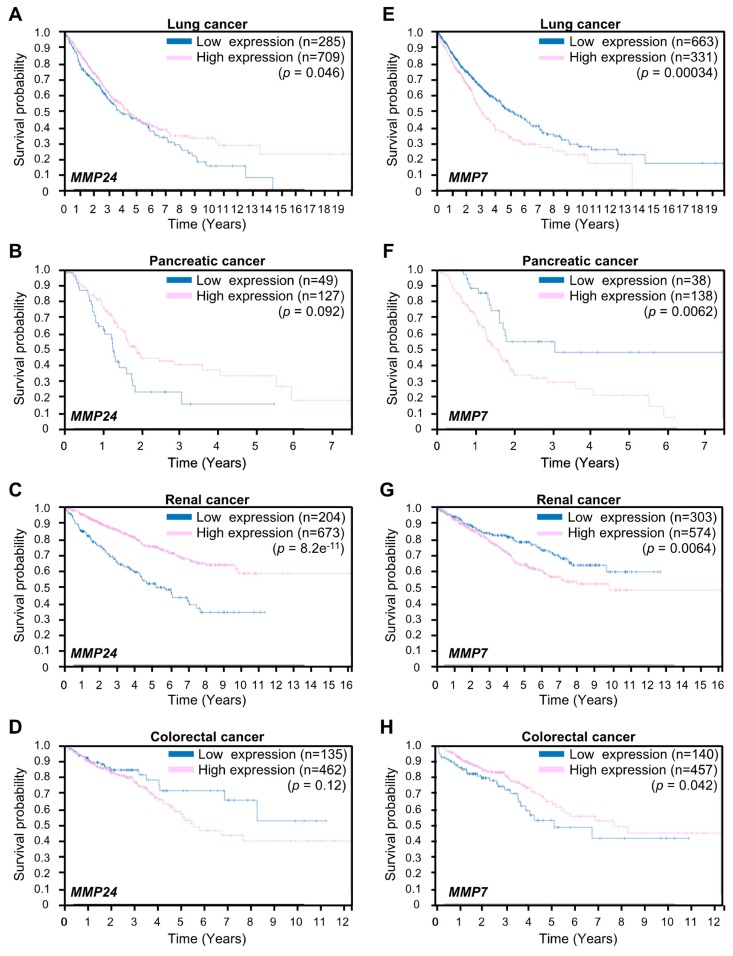
Expression of *MMP24* and *MMP7* in the prognosis of lung cancer, pancreatic cancer, renal cancer, and colorectal cancers. Kaplan–Meier plots for survival analysis of human lung (**A**,**E**), pancreatic (**B**,**F**), renal (**C**,**G**), and colorectal (**D**,**H**) cancer patients with high (magenta) and low (blue) *MMP24* (**A**–**D**) or *MMP7* (**E**–**H**) expression obtained from the Human Protein Atlas (version 19) available at www.proteinatlas.org. The links are: lung; https://www.proteinatlas.org/ENSG00000125966-MMP24/pathology/lung+cancer, https://www.proteinatlas.org/ENSG00000137673-MMP7/pathology/lung+cancer, pancreatic; https://www.proteinatlas.org/ENSG00000125966-MMP24/pathology/pancreatic+cancer, https://www.proteinatlas.org/ENSG00000137673-MMP7/pathology/pancreatic+cancer renal; https://www.proteinatlas.org/ENSG00000125966-MMP24/pathology/renal+cancer, https://www.proteinatlas.org/ENSG00000137673-MMP7/pathology/renal+cancer, colorectal; https://www.proteinatlas.org/ENSG00000125966-MMP24/pathology/colorectal+cancer, https://www.proteinatlas.org/ENSG00000137673-MMP7/pathology/colorectal+cancer.

We proceeded to examine whether expression of *MMP24* affects tumor aggressiveness. To this end, we investigated the correlation between *MMP24* expression and the survival rate in breast cancer patients using the Human Protein Atlas database (https://www.proteinatlas.org). The result showed that breast cancer patients with lower levels of *MMP24* expression exhibit worse survival rates overall (Figure 4). Further investigations revealed that also in other cancers including lung cancer, pancreatic cancer, and renal cancer, patients with lower levels of *MMP24* expression exhibit lower overall survival rates (Figure 5A–C). In contrast, although not statistically significant (*p* = 0.12; Figure 5D), there was a tendency that colorectal cancer patients with higher levels of *MMP24* expression exhibit lower survival rates. While the elastic modulus of tumor ECM is correlated with the aggressiveness of cancer cells [1,2,20,21], these results imply that in cancers such as breast cancer, lung cancer, pancreatic cancer, and renal cancer, MMP24 may retard tumor progression under the stiff environment of the cancer niche. 

Expression of *MMP7* encoding matrix metalloproteinase-7 (MMP7) is also increased in response to ECM stiffening [18]. MMP7 is known to promote cancer cell invasion [22,23]. We next examined the relationship between *MMP24* and *MMP7* expression with respect to tumor aggressiveness. In contrast to *MMP24*, higher levels of *MMP7* expression were associated with lower survival rates in patients with lung cancer, pancreatic cancer, or renal cancer but not colorectal cancer (Figure 5E–H), whereas *MMP7* was not a prognostic factor for breast cancer in the Human Protein Atlas database. Thus, *MMP7* could have a role opposite to that of *MMP24* in the regulation of aggressiveness of cancer cells.

## 4. Discussion

**Figure 6:**
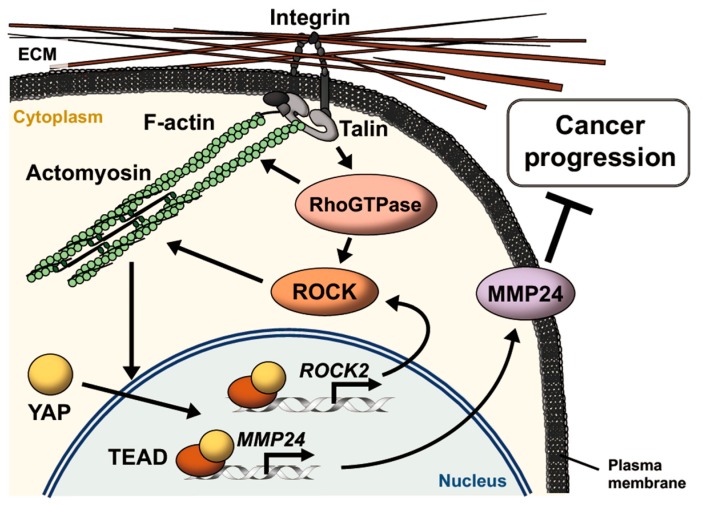
Schematic model for *MMP24* expression promoted by extracellular matrix (ECM) stiffening. Integrins bind to ECM and induce the formation of focal adhesions, leading to actin polymerization via the activation of Rho GTPases such as Rho, Rac, and Cdc42. Rho GTPases also induce activation of myosin via activation of ROCK. Actomyosin contraction enhances activation of this integrin signaling pathway via conformational change of the proteins as mechanosensors including integrins and talin in an ECM stiffness-dependent manner. Increase in the number of actin filaments promotes YAP nuclear translocation, resulting in induction of *ROCK2* and *MMP24* expression via TEAD activation. MMP24 expression attenuates the progression of various types of cancers such as breast cancer, lung cancer, and renal cancer.

Based on our results, we propose a model for hierarchical regulation of *MMP24* expression (Figure 6). Rigid substrates increase actomyosin contractility via YAP-dependent expression of *ROCK2*, which further activates YAP and TEAD. Activation of YAP-TEAD then promotes the expression of *MMP24*, retarding the progression of various types of cancers such as breast cancer, lung cancer, and renal cancer. 

YAP expression promotes the malignancy of cancer cells [24,25,26,27,28,29,30,31]. Two well-known YAP-TEAD target genes *CTGF* and *CCND1*, which encode connective tissue growth factor (CTGF) and cyclin D1, respectively, contribute to the progression of cancer [32,33,34]. *MMP24* expression that has been identified as a YAP-TEAD target gene in this study is believed to attenuate cancer progression, since high *MMP24* expression reflects prolonged overall survival rate of patients with cancers such as breast cancer, lung cancer, pancreatic cancer, and renal cancer (Figure 4 and Figure 5A–C). In these types of cancers, cancer aggressiveness would be promoted when expression of *MMP24* induced by YAP-TEAD is hampered. Thus, *MMP24* would be a prognostic factor in cancer patients and therefore, controlling *MMP24* expression could be a viable treatment strategy to block cancer progression.

In this study, we also showed that in contrast to *MMP24*, high expression of *MMP7*, which is another YAP-TEAD target gene, is associated with poor prognosis in lung cancer, pancreatic cancer, and renal cancer but not colorectal cancer (Figure 5E–H). It remains unclear why the role of *MMP24* in tumor aggressiveness is opposite to that of *MMP7* and their roles are cancer type-dependent. We also discovered that ECM substrates of MMP24 and MMP7 are different and that MMP7 expression promotes angiogenesis [35], which has a central role in tumor growth and metastasis [36], whereas the role of MMP24 in angiogenesis has not been clarified. The different roles of *MMP24* and *MMP7* in cancer progression could be due to the different ECM types surrounding the tumor [37], which control angiogenesis for metastasis and growth of cancer cells. Further studies are required for better understanding of the mechanism underlying cancer progression that involves the link between *MMP24* and *MMP7*.

## References

[1] Paszek M.J., Zahir N., Johnson K.R., Lakins J.N., Rozenberg G.I., Gefen A., Reinhart-King C.A., Margulies S.S., Dembo M., Boettiger D. (2005). Tensional homeostasis and the malignant phenotype. Cancer Cell.

[2] Levental K.R., Yu H., Kass L., Lakins J.N., Egeblad M., Erler J.T., Fong S.F., Csiszar K., Giaccia A., Weninger W. (2009). Matrix crosslinking forces tumor progression by enhancing integrin signaling. Cell.

[3] Mitra S.K., Hanson D.A., Schlaepfer D.D. (2005). Focal adhesion kinase: In command and control of cell motility. Nat. Rev. Mol. Cell Biol..

[4] Huveneers S., Danen E.H. (2009). Adhesion signaling - crosstalk between integrins, Src and Rho. J. Cell Sci..

[5] Plotnikov S.V., Pasapera A.M., Sabass B., Waterman C.M. (2012). Force fluctuations within focal adhesions mediate ECM-rigidity sensing to guide directed cell migration. Cell.

[6] Ebata T., Hirata H., Kawauchi K. (2016). Functions of the Tumor Suppressors p53 and Rb in Actin Cytoskeleton Remodeling. BioMed Res. Int..

[7] Dupont S., Morsut L., Aragona M., Enzo E., Giulitti S., Cordenonsi M., Zanconato F., Le Digabel J., Forcato M., Bicciato S. (2011). Role of YAP/TAZ in mechanotransduction. Nature.

[8] Low B.C., Pan C.Q., Shivashankar G.V., Bershadsky A., Sudol M., Sheetz M. (2014). YAP/TAZ as mechanosensors and mechanotransducers in regulating organ size and tumor growth. FEBS Lett..

[9] Ebata T., Mitsui Y., Sugimoto W., Maeda M., Araki K., Machiyama H., Harada I., Sawada Y., Fujita H., Hirata H. (2017). Substrate Stiffness Influences Doxorubicin-Induced p53 Activation via ROCK2 Expression. BioMed Res. Int..

[10] Sugimoto W., Itoh K., Mitsui Y., Ebata T., Fujita H., Hirata H., Kawauchi K. (2018). Substrate rigidity-dependent positive feedback regulation between YAP and ROCK2. Cell Adh. Migr..

[11] Nagase H., Visse R., Murphy G. (2006). Structure and function of matrix metalloproteinases and TIMPs. Cardiovasc. Res..

[12] Sounni N.E., Noel A. (2005). Membrane type-matrix metalloproteinases and tumor progression. Biochimie.

[13] Porlan E., Marti-Prado B., Morante-Redolat J.M., Consiglio A., Delgado A.C., Kypta R., Lopez-Otin C., Kirstein M., Farinas I. (2014). MT5-MMP regulates adult neural stem cell functional quiescence through the cleavage of N-cadherin. Nat. Cell Biol..

[14] Itoh Y. (2015). Membrane-type matrix metalloproteinases: Their functions and regulations. Matrix Biol..

[15] Yip A.K., Iwasaki K., Ursekar C., Machiyama H., Saxena M., Chen H., Harada I., Chiam K.H., Sawada Y. (2013). Cellular response to substrate rigidity is governed by either stress or strain. Biophys. J..

[16] Guo A.K., Hou Y.Y., Hirata H., Yamauchi S., Yip A.K., Chiam K.H., Tanaka N., Sawada Y., Kawauchi K. (2014). Loss of p53 enhances NF-kappaB-dependent lamellipodia formation. J. Cell. Physiol..

[17] Uhlen M., Zhang C., Lee S., Sjostedt E., Fagerberg L., Bidkhori G., Benfeitas R., Arif M., Liu Z., Edfors F. (2017). A pathology atlas of the human cancer transcriptome. Science.

[18] Nukuda A., Sasaki C., Ishihara S., Mizutani T., Nakamura K., Ayabe T., Kawabata K., Haga H. (2015). Stiff substrates increase YAP-signaling-mediated matrix metalloproteinase-7 expression. Oncogenesis.

[19] Zuberi K., Franz M., Rodriguez H., Montojo J., Lopes C.T., Bader G.D., Morris Q. (2013). GeneMANIA prediction server 2013 update. Nucleic Acids Res..

[20] Lopez J.I., Kang I., You W.K., McDonald D.M., Weaver V.M. (2011). In situ force mapping of mammary gland transformation. Integr. Biol..

[21] Gkretsi V., Stylianopoulos T. (2018). Cell Adhesion and Matrix Stiffness: Coordinating Cancer Cell Invasion and Metastasis. Front. Oncol..

[22] Basu S., Thorat R., Dalal S.N. (2015). MMP7 is required to mediate cell invasion and tumor formation upon Plakophilin3 loss. PLoS ONE.

[23] Chang M.C., Chen C.A., Chen P.J., Chiang Y.C., Chen Y.L., Mao T.L., Lin H.W., Chiang W.-H.L., Cheng W.F. (2012). Mesothelin enhances invasion of ovarian cancer by inducing MMP-7 through MAPK/ERK and JNK pathways. Biochem. J..

[24] Shen J., Cao B., Wang Y., Ma C., Zeng Z., Liu L., Li X., Tao D., Gong J., Xie D. (2018). Hippo component YAP promotes focal adhesion and tumour aggressiveness via transcriptionally activating THBS1/FAK signalling in breast cancer. J. Exp. Clin. Cancer Res..

[25] Lamar J.M., Stern P., Liu H., Schindler J.W., Jiang Z.G., Hynes R.O. (2012). The Hippo pathway target, YAP, promotes metastasis through its TEAD-interaction domain. Proc. Natl. Acad. Sci. USA.

[26] Lo Sardo F., Strano S., Blandino G. (2018). YAP and TAZ in Lung Cancer: Oncogenic Role and Clinical Targeting. Cancers.

[27] Su L.L., Ma W.X., Yuan J.F., Shao Y., Xiao W., Jiang S.J. (2012). Expression of Yes-associated protein in non-small cell lung cancer and its relationship with clinical pathological factors. Chin. Med. J..

[28] Yang S., Zhang L., Purohit V., Shukla S.K., Chen X., Yu F., Fu K., Chen Y., Solheim J., Singh P.K. (2015). Active YAP promotes pancreatic cancer cell motility, invasion and tumorigenesis in a mitotic phosphorylation-dependent manner through LPAR3. Oncotarget.

[29] Schutte U., Bisht S., Heukamp L.C., Kebschull M., Florin A., Haarmann J., Hoffmann P., Bendas G., Buettner R., Brossart P. (2014). Hippo signaling mediates proliferation, invasiveness, and metastatic potential of clear cell renal cell carcinoma. Transl. Oncol..

[30] Wang L., Shi S., Guo Z., Zhang X., Han S., Yang A., Wen W., Zhu Q. (2013). Overexpression of YAP and TAZ is an independent predictor of prognosis in colorectal cancer and related to the proliferation and metastasis of colon cancer cells. PLoS ONE.

[31] Zhao A.Y., Dai Y.J., Lian J.F., Huang Y., Lin J.G., Dai Y.B., Xu T.W. (2018). YAP regulates ALDH1A1 expression and stem cell property of bladder cancer cells. Onco Targets Ther..

[32] Zhu X., Zhong J., Zhao Z., Sheng J., Wang J., Liu J., Cui K., Chang J., Zhao H., Wong S. (2015). Epithelial derived CTGF promotes breast tumor progression via inducing EMT and collagen I fibers deposition. Oncotarget.

[33] Musgrove E.A., Caldon C.E., Barraclough J., Stone A., Sutherland R.L. (2011). Cyclin D as a therapeutic target in cancer. Nat. Rev. Cancer.

[34] Chu C.Y., Chang C.C., Prakash E., Kuo M.L. (2008). Connective tissue growth factor (CTGF) and cancer progression. J. Biomed. Sci..

[35] Huo N., Ichikawa Y., Kamiyama M., Ishikawa T., Hamaguchi Y., Hasegawa S., Nagashima Y., Miyazaki K., Shimada H. (2002). MMP-7 (matrilysin) accelerated growth of human umbilical vein endothelial cells. Cancer Lett..

[36] Fukuda A., Wang S.C., Morris J.P., Folias A.E., Liou A., Kim G.E., Akira S., Boucher K.M., Firpo M.A., Mulvihill S.J. (2011). Stat3 and MMP7 contribute to pancreatic ductal adenocarcinoma initiation and progression. Cancer Cell.

[37] Pickup M.W., Mouw J.K., Weaver V.M. (2014). The extracellular matrix modulates the hallmarks of cancer. EMBO Rep..

